# Mechanism of Fibronectin Binding to Human Trabecular Meshwork Exosomes and Its Modulation by Dexamethasone

**DOI:** 10.1371/journal.pone.0165326

**Published:** 2016-10-26

**Authors:** W. Michael Dismuke, Mikael Klingeborn, W. Daniel Stamer

**Affiliations:** 1 Department of Ophthalmology, Duke University, Durham, North Carolina, United States of America; 2 Department of Biomedical Engineering, Duke University, Durham, North Carolina, United States of America; Oregon Health and Science University, UNITED STATES

## Abstract

Exosomes are emerging as important mediators of cell-matrix interactions by means of specific adhesion proteins. Changes in the tissue-specific exosomal protein expression may underlie pathological conditions whereby extracellular matrix turnover and homeostasis is disrupted. Ocular hypertension due to extracellular matrix accumulation in the trabecular meshwork is a hallmark of glucocorticoid-induced glaucoma. In the trabecular meshwork, exosomal fibronectin mediates cell matrix interactions at cellular structures called “invadosomes”. Trabecular meshwork cells use invadosomes to turn over their surrounding matrix and maintain passageways for flow of aqueous humor. In this study, we observed that human trabecular meshwork explants treated with dexamethasone released exosomes with significantly reduced amounts of fibronectin bound per exosome. Further, we found that exosome-fibronectin binding is heparan sulfate-dependent, consistent with our observation that trabecular meshwork exosomes are enriched in the heparin/heparan sulfate binding annexins A2 and A6. In this way, dexamethasone-treated explants released exosomes with a significant reduction in annexin A2 and A6 per exosome. Interestingly, we did not detect exosomal matrix metalloproteinases, but we identified abundant dipeptidyl peptidase 4, a serine protease whose activity was reduced on exosomes isolated from dexamethasone-treated explants. Together, our findings demonstrate mechanistically how corticosteroid-induced alterations in exosomal adhesion cargo and properties can account for the pathological matrix accumulation seen in many glaucoma patients.

## Introduction

Elevated intraocular pressure (IOP) is the primary risk factor for developing glaucoma, a leading cause of irreversible blindness worldwide [1]. An idiopathic increase in resistance to aqueous humor drainage through the trabecular meshwork (TM) and Schlemm’s canal in the conventional outflow pathway, causes ocular hypertension [2]. The precise mechanisms that control outflow resistance of the TM are unclear but extracellular matrix (ECM) turnover is thought to be critically important in maintaining healthy IOP [3]. Further, aberrant accumulation of ECM is a characteristic of two major forms of glaucoma, primary open-angle glaucoma and corticosteroid-induced glaucoma [4, 5]. Currently there is no cure for glaucoma and the only effective therapy is lowering IOP [6]. Unfortunately, no clinically available IOP lowering drugs directly target the conventional drainage tissues or address the pathological ECM changes thought to impede drainage.

Turnover of ECM in the TM is vigorous, with some protein half-lives being less than two days [7]. TM cells form highly dynamic, actin-rich organelles called “invadosomes” [8, 9]. In the TM and other tissues these structures are enriched with matrix metalloproteases (MMPs) and act as sites of focal ECM degradation and remodeling [10]. Inhibition or addition of MMPs to perfused human eye models, decreases or increases outflow respectively, demonstrating the critical role matrix degradation plays in regulating normal TM function [11]. However, MMPs are just one important component of these organelles contributing to the complex process of matrix remodeling. Recently, extracellular nanovesicles called exosomes have been localized to invadosome-like structures [12] and shown to be critical in the cell-specific invadosome function, such as invasiveness and migration of cancer cells, who also actively turnover ECM [13].

Exosomes are released from TM cells in culture [14–16], yet their function in outflow physiology is unknown. Proteomic analysis of TM cell exosomes showed that fibronectin (Fn) is an abundant component [16]. This is consistent with a number of reports which have shown exosomal binding of Fn using a number of methods such as flowcytometry[17], capture of exosomes on immobilized Fn[13] and density gradient ultracentrifugation[18, 19]. These results show exosomal affinity for Fn may be a common property of exosomes shared across a number of cell types. Recently, exosomes have been implicated in a number of cell-ECM interactions, including several that depend on exosomal Fn [18, 20]. These data suggest exosome-bound Fn plays an important role in cell-matrix interactions. Additionally, the amount of Fn bound to exosomes influences the magnitude of the cell-matrix interaction. For example, decreasing the amount of exosomal Fn decreases cell motility [18].

Experimentally, secretion of Fn from TM cells is dramatically elevated upon treatment with corticosteroids [21]. In fact, treatment of TM cells and tissues with corticosteroids has been used to identify the first glaucoma gene, MYOC [22], and as a reliable model to study ECM turnover by the TM in a controlled fashion. Here we used corticosteroid treatment of TM explants to determine whether released exosomes are altered by this glaucoma-inducing compound. We found that despite a Dexamethasone (Dex)-induced increase in Fn secretion by TM explants, the released exosomes had less bound Fn. Additionally, we show that TM exosome-Fn binding is dependent on the presence of the glycosaminoglycan heparan sulfate. Finally, we found that TM exosomes are enriched in the known heparin/heparan sulfate binding proteins annexin A2 and A6 [23, 24], and that Dex treatment of the TM explants significantly reduced the amount of these annexins per exosome. Our findings are the first to demonstrate that a drug causing ECM accumulation in the TM [4], ocular hypertension [25] and glaucoma in humans and animal models, alters exosomal cargo and properties in a way that can account for the observed ocular phenotype.

## Materials and Methods

### Human TM explants and cell strains

De-identified human donor whole globes or corneal rims (following corneal button removal for transplant) were obtained from donors who gave consent for research (Miracles In Sight, Winston-Salem, NC) and the trabecular meshwork was isolated using a blunt dissection technique. For experiments involving dexamethasone treatment, the anterior portion of the eye tissue was bisected in a random orientation to yield two equal portions, prior to TM removal. Each portion of the TM explant tissue was placed in one well of a 24-well plate in low glucose DMEM (Dulbecco’s modified Eagles’s medium), containing 1% exosome-depleted FBS (fetal bovine serum), 100 U/ml penicillin, 0.1mg/ml streptomycin and 0.29mg/ml glutamine and maintained in humidified air containing 5% CO_2_ at 37°C for 24 hours prior to experimental treatments. Human TM cells were isolated from donor eyes using a blunt dissection technique followed by an extracellular matrix digestion protocol. Cell stains were characterized as previously described [26].

### Dexamethasone treatment of TM explants

Following the initial 24 hour culture period, explants were washed in DPBS (Dulbecco’s phosphate buffered saline) and fresh culture media containing vehicle (ethanol, 0.1% v:v) or dexamethasone (100nM) was added. Explants were cultured for 72 hours in humidified air containing 5% CO_2_ at 37°C. Afterward, media was collected and stored at -20°C prior to exosome purification. TM explants were blotted dry, weighed and frozen at -80°C.

### Exosome isolation

Three methods for exosome isolation were used for these studies. 1) For the majority of experiments, exosomes were isolated by precipitation as follows: Conditioned media was spun at 3000 *g* for 15min and the cleared supernatant was transferred to a clean tube. Precipitation solution (50% PEG8000, 0.5M NaCl in DPBS) was added to the cleared supernatant (1:6 v:v, for a final [PEG8000] = 8.3%), mixed by repeated inversion and left overnight at 4°C. The next day tubes were centrifuged at 1500 *g* for 30min to pellet exosomes and the supernatant was carefully removed. Tubes were centrifuged again at 1500 *g* for 5min and remaining supernatant was removed. Pelleted exosomes were resuspended in DPBS for further analysis. 2) For experiments involving addition of FBS or Fn to conditioned media or heparanase digestion, exosomes were prepared by serial ultracentrifugation as follows. Conditioned media was centrifuged at 3000 *g* for 15 min to pellet debris. Cleared supernatant was transferred to fresh ultracentrifuge tubes. In some experiments, purified human plasma fibronectin (#F0895, Sigma-Aldrich) or exosome-depleted FBS (#S11550, Atlanta Biologicals, Lawrenceville, GA; FBS spun overnight at 100,000 *g*) was added to cleared supernatant for a final concentration of 2.5 μg/ml or 10% v:v, respectively. Cleared supernatant was spun at 10,000 *g* for 40min at 4°C with a Beckman SW28 rotor. The resulting supernatant was removed, transferred to another ultracentrifuge tube and centrifuged at 100,000 *g* for 70min at 4°C (SW28 rotor). Next the supernatant was discarded. The resulting pellet was either resuspended in DPBS and directly used in subsequent centrifugation or for experiments involving digestion of heparan sulfate, DPBS ± heparanase II [50U/ml final](#H6512, Sigma-Aldrich) was added to pellets and incubated at 34°C for 1hr prior to subsequent centrifugation. Finally, resuspended pellets were centrifuged again at 100,000 *g* for 70min at 4°C (SW28 rotor). The supernatant was carefully removed with a pipette, the tube walls were wiped clean and the pellet was resuspended in 4x Laemmli/5% BME. 3) For experiments to determine vesicle density, conditioned media (CM) was centrifuged at 3000 *g* for 15min. Cleared supernatant was then concentrated using 100,000 MWCO PES Spin-X UF Concentrator (Corning) following the manufactures instructions. Concentrated CM was mixed with 60% OptiPrep (#D1556, Sigma-Aldrich) to a final concentration of 40% and placed in the bottom of a TLS-55 ultracentrifuge tube. Thirty, 20 and 10% OptiPrep solutions were carefully layered on top of the 40% solution and centrifuged at 100,000 *g* for 17hrs at 4°C. Fractions were collected using a pipette and weighed to determine density.

### Nanoparticle tracking analysis

Vesicle diameter and concentration were determined using nanoparticle tracking analysis (NTA)(ZetaView, 405nm laser; Particle Metrix, Meerbusch, Germany) calibrated with polystyrene beads. For analysis, samples were diluted in DPBS. Measurements from 11 positions within the imaging chamber at 24°C were used to determine sample vesicle size and concentration.

### Western blots

Exosome samples solubilized in Laemmli buffer were loaded onto SDS-PAGE gels by equal volume or equal particle number[18], separated electrophoretically and transferred to nitrocellulose. Membranes were blocked with 10% fat-free milk/water for 30min at room temp on a rocking platform. Blocking solution was exchanged with primary antibody solutions (antibodies diluted in 5% fat-free milk/TBST) and incubated on a rocking platform for 4hrs at room temperature or overnight at 4°C. Primary antibodies used were as follows: anti-Fn (#sc8422, Santa Cruz), anti-annexin A2 (#610068, BD Transduction Laboratories), anti-annexin A6 (#ab52221, Abcam), lactadherin (MFG-E8)(#sc271574, Santa Cruz), myocilin (custom polyclonal [27]). Primary antibodies were removed and blots were washed 3 times, for 5 minutes with TBST. Antibody solutions containing secondary, HRP-conjugated antibodies (goat anti-mouse HRP # 115035146, goat anti-rabbit HRP # 111035144, Jackson ImmunoResearch, West Grove, PA) were added and incubated for 1hr at room temperature while rocking. Blots were then washed 3 times for 5 min in TBST and developed using chemiluminescent reagents (HyGLO; #E2400, Denville Scientific, South Plainfield, NJ) and X-ray film (#30–101, Genesee Scientific, San Diego, CA).

### Dot blots

TM cell conditioned media (collected over 2.5hrs, serum-free) was centrifuged at 3000 *g* for 15min then split equally seven ways. Purified heparan sulfate (#H7640, Sigma-Aldrich) was added in increasing concentrations to the media. Tubes containing this conditioned media±heparan sulfate were then incubated on a rocker for 1hr at room temperature. Exosomes were isolated by precipitation as described. Resulting exosome pellets were resuspended in PBS. Nitrocellulose membrane was wetted for 30min in TBS then placed into a dot-blot apparatus (Bio-Rad). The membrane was washed per manufacturer’s instructions. Exosome samples were added to the wells of the apparatus and incubated under low vacuum for 30min, followed by full vacuum and washed 3 times with TBS. The membrane was removed and dried for 1hr. Next the membrane was re-wetted with TBS then blocked in 10% fat-free milk/water for 30min at room temp on a rocking platform. Blocking solution was exchanged with Anti-Fn antibody (#sc8422, Santa Cruz) solution (antibodies diluted in 5% fat-free milk/TBST) and incubated on a rocking platform for 4hrs at room temperature. Primary antibodies were removed and blots were washed 3 times, for 5min with TBST. Secondary antibody solution (goat anti-mouse HRP #115035146, Jackson ImmunoResearch) were added and incubated for 1hr at room temperature while rocking. Membranes were then washed 3 times for 5min in TBST and developed using chemiluminescent reagents (HyGLO) and X-ray film (Genesee Scientific).

### LC-MS/MS

Serum-free conditioned media from human TM cells was collected and exosomes were prepared as described. Exosomes were resuspended in DPBS with 0.1% and centrifuged at 100,000xg for 1hr to pellet insoluble proteins. The resulting supernatant was incubated on a rotator with Heparin Sepharose 6 Fast Flow beads overnight at 4°C. The beads were then washed 3x with DPBS and incubated with 13ng/μL of Trypsin Gold (Promega Corporation) in 10mM ammonium bicarbonate overnight at 37°C. The digest solution was vacuum dried (Savant SpeedVac) And digested peptides were processed, identified and quantified by LC-MS/MS as previously described[28].

### Densitometric analysis

X-ray films from exposures where all bands appeared to be within the dynamic range of the film were scanned (Canon LiDE 210 scanner). Band intensity was determined using NIH ImageJ gel analysis tools. For comparisons of band intensity where samples were loaded by equal volume, measured band intensity was normalized to the number of particles (determined by NTA) per lane. For experiments where an equal number of particles were loaded per lane, band intensities were directly compared.

## Results

### Human trabecular meshwork tissue releases exosomes

**Fig 1:**
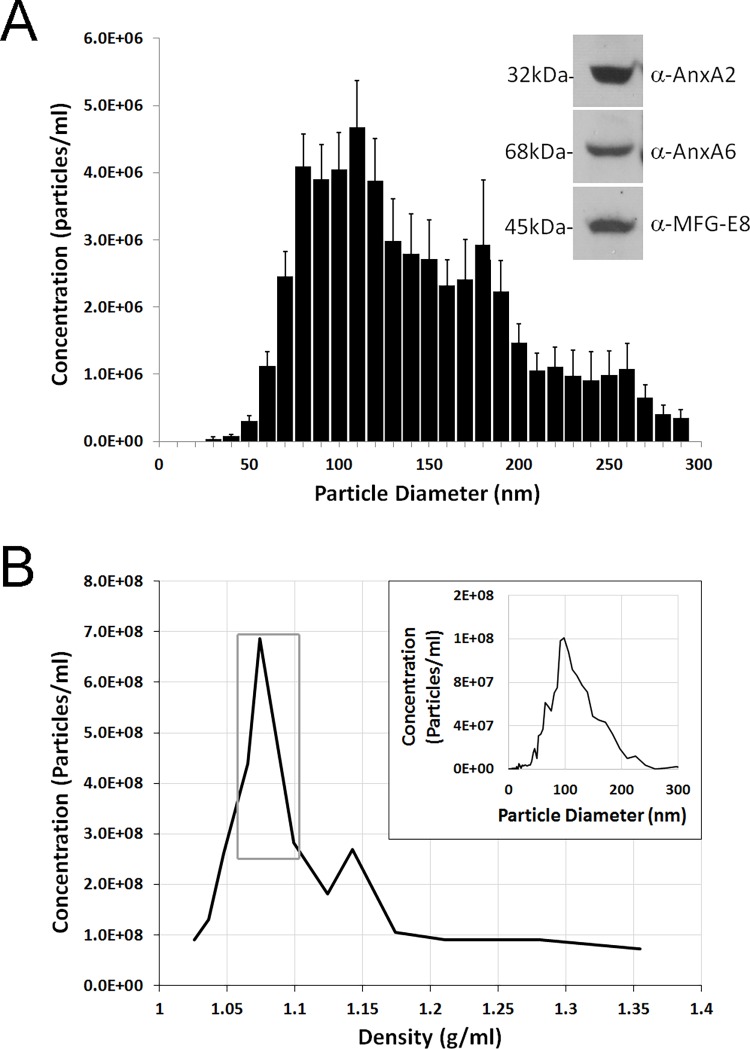
Human trabecular meshwork (TM) explants release exosomes. (A) TM explants from six human donor eyes were cultured separately for 72 hours. Extracellular vesicles (EVs) were prepared from the conditioned media and analyzed for size by nanoparticle tracking analysis (NTA) and protein content by western blot (inset). NTA data is binned in 10 nm increments and represent the mean±SD of six individual biological replicates. Western blots are representative of the results obtained from the six individual biological replicates. AnxA2, Annexin 2; AnxA6, Annexin 6; MFG-E8, lactadherin. (B) A single human TM explant was cultured for 9 days (media collected every 3 days). This media was concentrated by ultrafiltration and mixed into the bottom portion of an Iodixanol (OptiPrep) density gradient and ultracentrifuged overnight. Fractions were collected, their density was determined and the concentration of EVs in each fraction was determined by NTA. Fractions corresponding to the density of exosomes (1.07–1.10 g/ml, gray box) were combined and analyzed for size distribution by NTA (inset).

Cultured human TM cells release extracellular vesicles (EV) with properties consistent with exosomes [16]. However, EV release from human TM cells resident in intact tissue has never been shown. To address this, EVs were prepared from the conditioned media of TM explants dissected from human donor eyes. The resulting EVs had a size consistent with exosomes (Fig 1A). Compared to other types of EVs, exosomes are reported to be uniquely enriched with tetraspanins including the annexins and the phosphatidylserine binding protein lactadherin [29]. Western blots show that the TM explant derived EVs contain annexin A2, A6 and lactadherin (MFG-E8) (Fig 1A inset). EVs can be differentiated by their density [30]. Thus, EVs released from the human TM explants, were analyzed after floatation into a continuous iodixanol (OptiPrep) density gradient. Gradient fractions were collected and nanoparticle concentrations were quantified by nanoparticle tracking analysis. The majority of the TM explant EVs ranged between 1.07–1.10 g/ml (Fig 1B), which is a density consistent with exosomes [31, 32]. Additionally, the vesicles within this density range had a size distribution identical to exosomes (diameter: mean = 104 nm, mode = 99 nm) (Fig 1B inset). In summary, these data demonstrate for the first time, based on currently accepted criteria, that exosomes are produced in an organotypic culture model of the TM.

### Dexamethasone decreases the amount of bound fibronectin to exosomes

**Fig 2:**
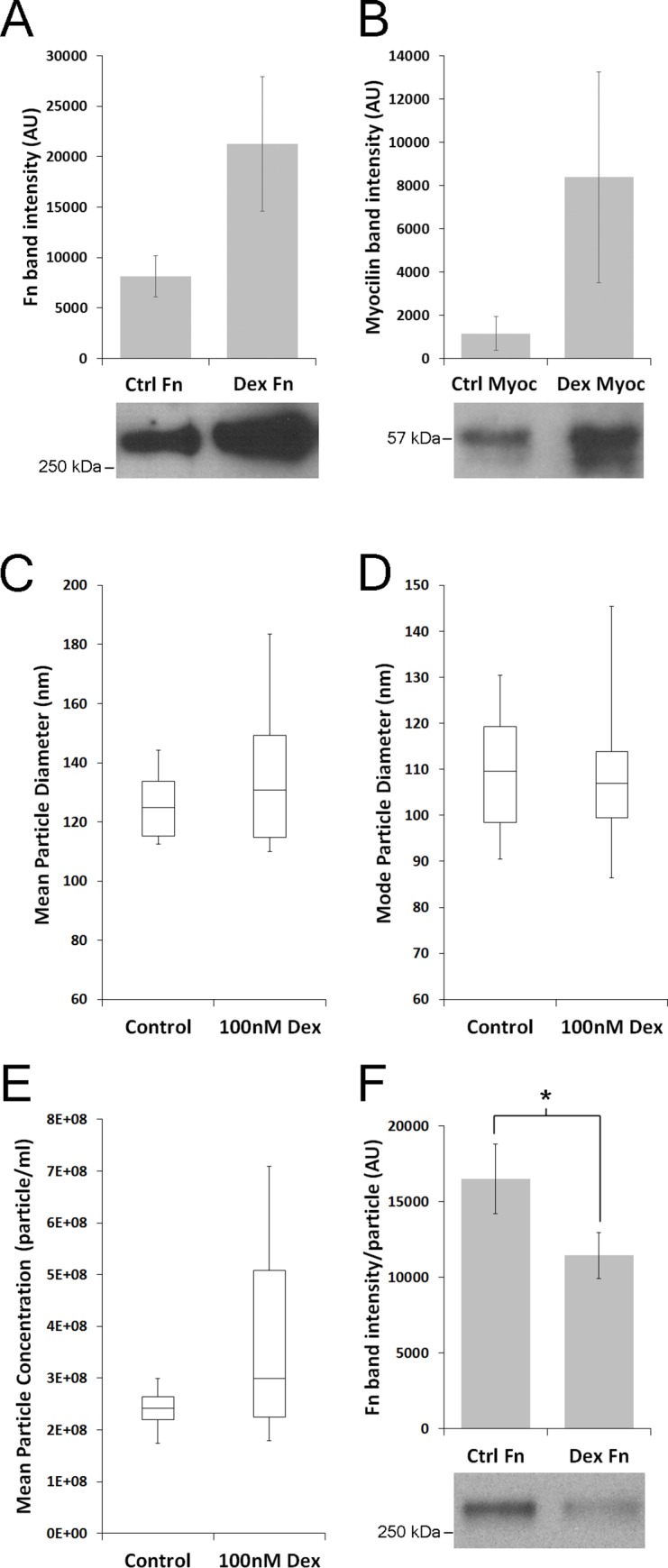
Treatment of human trabecular meshwork (TM) explants with dexamethasone (Dex) reduces the amount of exosome bound fibronectin (Fn). TM explants were dissected from human donor eyes and split in half. Each half was cultured for 72 hours in control (Ctrl) media or media containing 100nM dexamethasone (Dex). Conditioned media from control and Dex treated TM explants was probed for fibronectin (Fn) and myocilin (Myoc) by western blot. Band intensity was normalized to dry TM tissue weight. Dex treatment increased the secretion of (A) Fn and (B) Myoc in the conditioned media (mean ± SD, n = 3). Exosomes were isolated, sized and counted by nanoparticle tracking analysis. (C) Dex treatment does not alter the mean or (D) mode size of exosomes released by human TM explants (control n = 6, Dex n = 6). (E) Dex treatment does not significantly alter the amount of exosomes in the conditioned media (p = 0.1654, Student’s t-test, n = 6). (F) Equal volumes of exosomes were analyzed for Fn content by western blot from six donors (6 control, 6 Dex treated). Fn band intensity was determined using ImageJ and the intensity was normalized to the number of exosomes loaded per lane. The representative bands shown are from a single donor, control = 3.39E+08 particles and Dex = 3.49E+08 particles. Bar graph is the mean±SEM of six control and Dex treated Fn band intensities normalized to the number of particles per lane. *p<0.05, control vs Dex, n = 6, Student’s t-test. Box-and-whisker plots in A, B and C are the max, 3^rd^ quartile, median, 1^st^ quartile and min.

Exosomes isolated from a number of biological fluids and cell types (including human TM cells) are bound to fibronectin (Fn) [16, 33]. TM cells secrete fibronectin and increase its secretion in response to corticosteroid treatment. To determine whether exosomes from TM explants also bind fibronectin and whether Dex treatment impacts binding, we split TM tissue from a single donor eye into two equal portions (to control for known donor variability in corticosteroid-responses [34]) and cultured them separately in the presence or absence of dexamethasone (Dex) (100 nM) for 3 days. Western blots show that human TM explants responded to Dex treatment like TM cells *in vitro* [35], with increases in Fn and myocilin (Myoc) secretion (Fig 2A and 2B). Because of this increase in Fn secretion we hypothesized that Dex treatment of human TM explants will increase the amount of Fn bound to released exosomes compared to exosomes from untreated explants. In the six individual donor tissues tested, Dex did not significantly affect the mean or mode vesicle size (Fig 2C and 2D). While there was a slight increase in mean vesicle concentration in the Dex treated samples, the change varied from donor to donor and was not significant (Fig 2E). Exosomes from control and Dex treated TM explants were analyzed for Fn content by western blot. Contrary to our hypothesis and despite the variability between donors, there was a significant decrease in the amount of Fn detected per exosome (Fig 2F).

### TM exosomes bind fibronectin on their external surface

**Fig 3:**
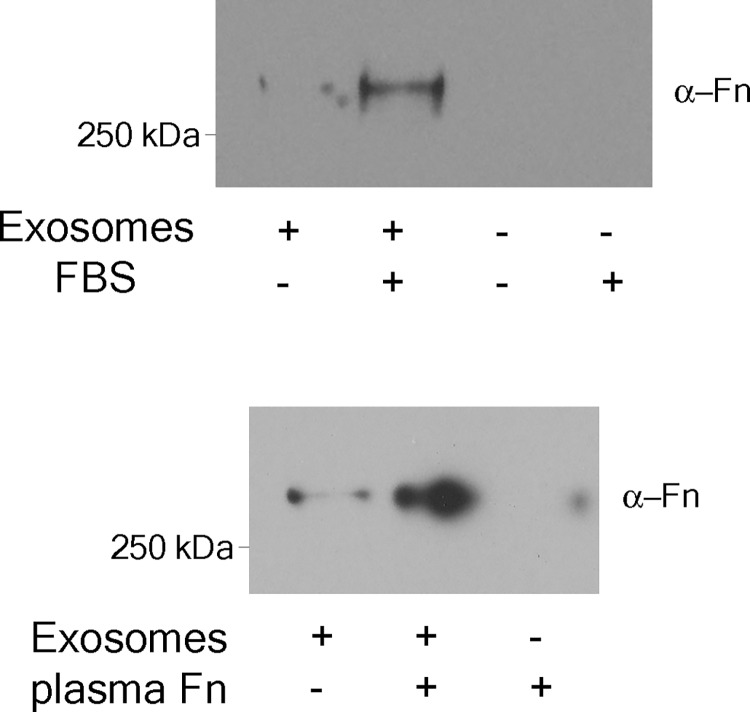
Trabecular meshwork (TM) exosomes bind fibronectin on the external surface. Serum-free medium was conditioned for 2.5 hours with primary TM cell cultures. Conditioned media was split in half and either exosome-depleted FBS (10% final v:v) or purified human plasma fibronectin (Fn, 2.5μg/ml [final]) was added to one portion. Exosomes were prepared from both portions (and from unconditioned media + FBS or purified Fn) and Fn content was assessed by Western blot. The blots shown represent the results from a minimum 3 biological replicates (cell strains isolated from different human donors).

To better understand what changes occurred from Dex treatment that affect the Fn binding capacity of the exosomes and perhaps account for the ECM accumulation in steroid-induced glaucoma, we investigated the binding of Fn to TM exosomes *in vitro*. Exosomes were prepared from serum starved cells and probed for Fn abundance. Only trace amounts of Fn were detected (Fig 3). However, when TM conditioned media (serum-free) was incubated with FBS or purified Fn and exosomes were prepared, exosomes now pelleted with bound Fn. Since neither FBS nor purified Fn alone pellet under identical conditions, we conclude that the TM cell exosomes are released without Fn but have the capacity to bind it once outside their parent cells.

### Heparan sulfate-dependent binding of fibronectin to TM exosomes

**Fig 4:**
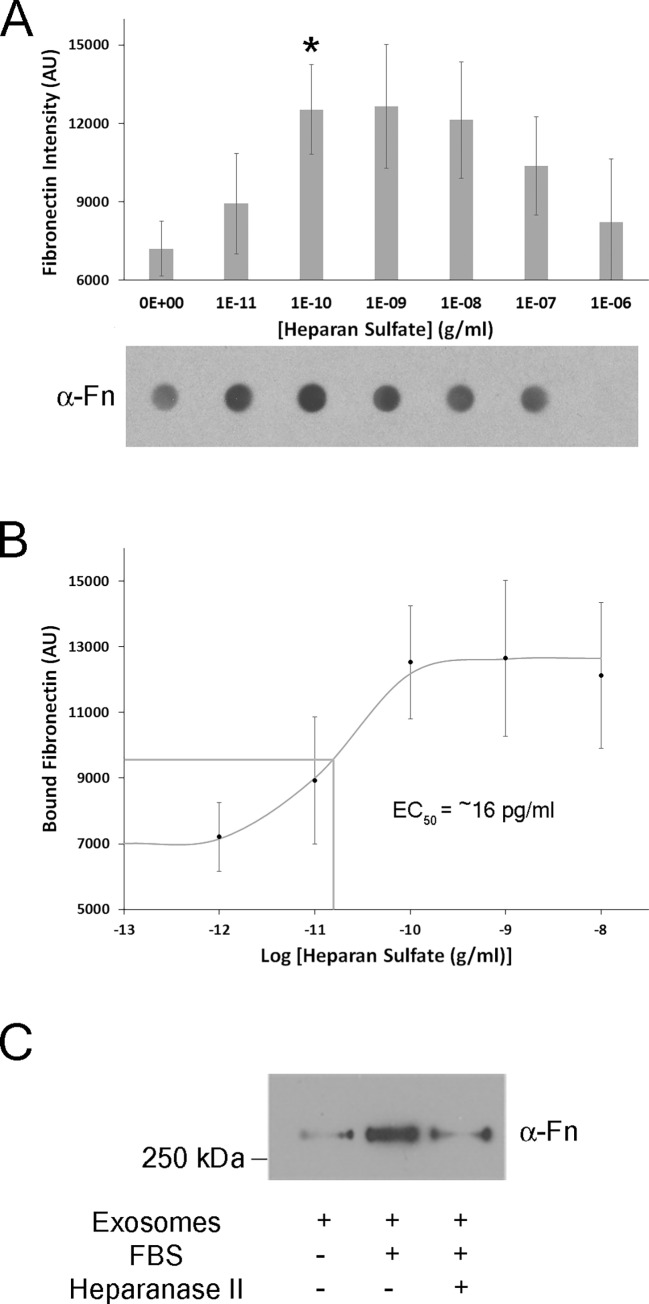
Molecular interaction between fibronectin and heparan sulfate on trabecular meshwork (TM) exosome surfaces. (A) Serum-free media conditioned by human TM cells for 2.5 hours was split into 7 equal portions and purified heparan sulfate was added at increasing log_10_ concentrations. Mixtures were incubated for 1 hour on a rocker at room temperature. Exosomes were then isolated from each sample and Fn content was assessed by dot blot. The intensity of Fn staining from 3 biological replicates (cell strains isolated from 3 different human donor eyes) were measured with ImageJ. The bar graph data is the mean±SEM of the 3 replicates. One representative dot blot is shown underneath. *p<0.05, [heparan sulfate] vs no heparan sulfate, Student’s t-test. (B) A dose-response curve was generated from the data shown in A (3 biological replicates). When no exogenous heparan sulfate was added, an endogenous concentration on the exosome of 1pM was used to generate the curve and estimate an EC_50_ value. **C,** Conditioned media was split into 3 equal portions. Exosome-depleted FBS (10% final v:v) was added to two portions. Exosomes were prepared, resuspended in PBS with or without a bacterial heparanase II and incubated at 34°C for 1 hour with periodic shaking (approx. 10 minute intervals). Fn content of exosomes was assessed by western blot. Blot shown is representative of 3 biological replicates from cell strains isolated from different donor eyes.

Recently, exosomes were shown to bind Fn via the heparan sulfate proteoglycan, syndecan-1[17]. While no syndecans were detected in the TM exosome proteome, a secreted heparan sulfate proteoglycan, perlecan was observed [16]. To test whether a receptor for heparan sulfate exists and mediates Fn binding on TM exosomes, we incubated conditioned media from TM cell monolayers with increasing concentrations of heparan sulfate, isolated exosomes from the conditioned media and probed for Fn. Results show a concentration-dependent increase in exosome-bound Fn, attaining maximal binding and statistical significance at 100 pg/ml heparan sulfate (Fig 4A). Interestingly, Fn-exosome binding was biphasic, first increasing, then decreasing dose-dependently at higher heparan sulfate concentrations. In these experiments, the only Fn source for exosome binding was Fn secreted from the TM cells into the serum free media over 2.5 hrs. In separate experiments, this basal Fn concentration was measured to be 0.24±0.03 μg/ml (mean±SD). Using this data, we constructed a dose-response curve that allowed us to estimate an EC_50_ value of ~16 pg/ml heparan sulfate at the given Fn concentration (Fig 4B). To further test whether heparan sulfate mediates the exosome-Fn binding, a bacterial heparanase was shown to remove exosome-bound Fn (Fig 4C) similar to previously reported [17]. These data confirm that TM exosomes are released with a surface receptor for heparan sulfate that mediates the binding of Fn to the exosomes.

### Identification of potential heparin-binding exosomal proteins

To identify exosomal proteins capable of binding heparin/heparan sulfate we purified exosomes from human TM cells. Exosomes were lysed in 0.1% SDS, centrifuged to remove insoluble proteins and the resulting supernatant was incubated with heparin sepharose beads. The beads were washed, and bound proteins were digested with trypsin for identification by LC-MS/MS. Of the 4 proteins identified in both biological replicates, two known heparin binding proteins were identified; fibronectin and Annexin A2[23] (Table 1). The remaining identified proteins are cytoskeletal, not known to bind heparin and likely represent contaminants. This data demonstrates that annexin A2 on TM exosomes is the most likely candidate for an exosomal heparin receptor.

**Table 1:** List of TM exosomal proteins bound to heparin beads identified by mass-spec in two biological replicates.

Protein	# of total spectra from each replicate	# of unique peptides from each replicate	% coverage from each replicate
Fibronectin	60, 39	55, 37	23, 17
Vimentin	29, 11	25, 11	52, 23
Annexin A2	8, 6	8, 6	28, 19
Actin	8, 6	1, 2	25, 15

### Dexamethasone decreases the expression of exosomal heparin-binding annexins

**Fig 5:**
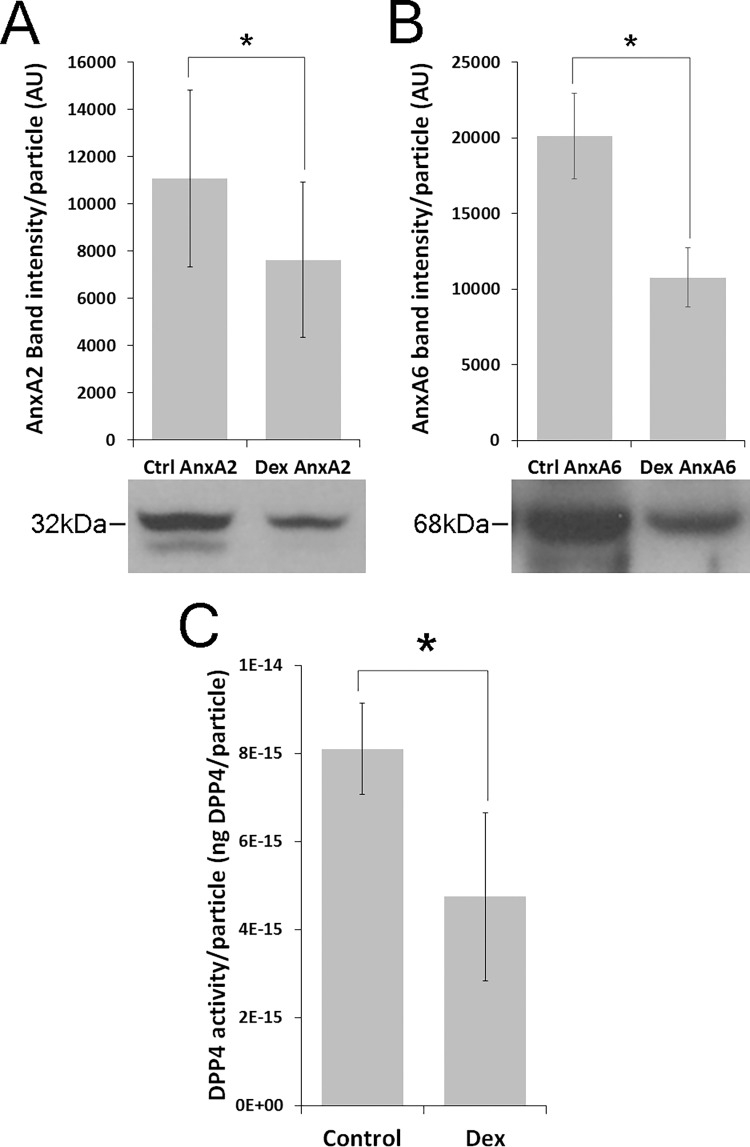
Treatment of human trabecular meshwork (TM) explants with dexamethasone decreases the amount of exosomal annexins A2/A6 and dipeptidyl peptidase activity. Exosomes were prepared from media conditioned by TM explants and analyzed for the presence of the heparin/heparan sulfate binding proteins (A) annexin A2 and (B) annexin A6. Intensity of immunoblot staining was measured with ImageJ, normalized to the number of vesicles loaded per lane and shown in bar graphs as mean±SEM. Panel A is representative of 4 biological replicates (equal particle loading per lane, 4 control, 4 Dex treated) and panel B is representative of 6 biological replicates (6 control, 6 Dex treated). *p<0.05 control vs Dex, Student’s t-test; annexin A2 n = 4, annexin A6 n = 6. **C,** Equal amounts of exosomes from control and Dex treated TM explants were analyzed for DPP4 activity using a peptide cleavage assay. Measured activity was fitted to a standard curve generated with known amounts of recombinant DPP4. Data shown as the mean±SD of 4 biological replicates with technical measurements in triplicate. *p<0.05 control vs Dex, Student’s T-test, n = 4.

Since treatment of human TM explants with Dex decreases Fn bound to released exosomes and exosome-Fn binding is dependent on heparan sulfate, we next tested whether exosomes from Dex treated TM explants showed decreases in the heparin/heparan sulfate binding annexins A2 and A6 [23, 24]. Exosomes from Dex treated human TM explants had significantly lower amounts of both annexin A2 and A6 by immunoblot compared to exosomes from untreated explants (Fig 5A and 5B). Since binding of Fn to TM exosomes is mediated through heparin/heparan sulfate, we conclude that a reduction in heparin/heparan sulfate binding proteins likely contributes to decreased Fn binding in exosomes from Dex treated explants.

### Dexamethasone decreases exosomal dipeptidyl peptidase activity

Fn-bound exosomes are reported to mediate a number of cell-matrix interactions including migration and invasion [18, 20], two processes dependent on podosomes/invadopodia and ECM digestion [10]. Since Dex treatment of TM decreases MMP secretion/activity [35, 36] and accumulation of matrix material [4], we next tested whether Dex altered exosomal protease activity. In our initial studies, we failed to detect exosome-specific MMP activity using an insolution fluorescent gelatinase assay. However, proteomic analysis of TM exosomes, identified the serine protease dipeptidyl peptidase-4 (DPP4) [16]. DPP4 localizes to invadopodia and its inhibition results in reduced invasiveness [37, 38]. When examined specifically, we observed that exosomes released from Dex treated TM explants had significantly reduced levels of DPP4 activity compared to untreated controls (Fig 5C). A reduction in DPP4 activity with Dex treatment is consistent with the resulting ECM dysregulation and accumulation in the TM of patients with steroid induced ocular hypertension.

## Discussion

Like primary TM cells in culture, we show that explants of the human trabecular meshwork in organ culture produce exosomes. Dexamethasone treatment of explants, which can alter TM function and cause glaucoma, significantly reduced Fn on exosomal surfaces. Mechanistically, we show that TM exosome-Fn binding is dependent on heparan sulfate. Significantly, two heparin/heparan sulfate binding annexins plus the peptidase DPP4 are reduced upon treatment of explants with Dex. Taken together, these data support a mechanism by which Dex negatively impacts exosome-mediated binding of ECM for homeostatic degradation by TM cells.

In the TM, impaired matrix degradation results in decreased aqueous humor drainage which can lead to elevated intraocular pressure, the primary risk factor for developing glaucoma [6]. Supporting this notion, increased ECM accumulation is a common hallmark of TM tissue from glaucomatous patients [4]. Impaired ECM turnover in glaucomatous TM suggests that the mechanisms to interact with and modify surrounding ECM are dysfunctional. Cells interact with their surrounding matrix through several cellular structures range from migratory podosomes to invasive invadopodia, which fall under the general category of “invadosomes” [10]. While these structures perform different functions in cells, they appear to utilize interconnected processes. With that in mind, exosomes have been shown to perform essential functions in a number of these structures. For example, exosomes are released at and promote the formation of invadopodia [12, 13]. Exosomes also promote the assembly of focal adhesions [39] and control the rapidity and directionality of cell migration [18, 20]. The later phenomenon being due to Fn bound to the exosome surface that may help match matrix ligands to their complimentary cell surface receptors to coordinate invadosome functions [18]. Since exosomes bound to Fn is an essential component of invadosomes, our data support a mechanism by which a similar scenario occurs for invadosomes in TM cells. Thus, Dex-induced reduction in exosome bound Fn provides in part an explanation for the accumulation of ECM material in the TM of Dex-induced animal models of ocular hypertension and patients with steroid induced glaucoma.

Several receptors mediate Fn binding to exosomes including heparan sulfate proteoglycans and integrins [17, 40, 41]. Our data support an additional mechanism by which Fn binds to exosomes in a complex of heparan sulfate and annexin proteins. When we purified TM exosomes, we found a number of proteins of the annexin family of tetraspanins, which are known to be enriched on exosomes [29] and thought to be located on the exosomal surface[42]. From TM exosomes, we detected abundant expression of the HS/heparin-binding annexins A2 and A6 [23, 24]. The idea that exosomes are released with receptors for heparin/heparan sulfate on their surface is supported by the recently described method of exosome purification via heparin affinity column [43]. Since it is well known that Fn binds heparin/heparan sulfate, we propose that exosomal annexins coordinate binding of heparin/heparan sulfate and Fn to TM exosomes. In the present study, we were able to alter Fn binding to exosomes in three ways: First, Dex treatment reduced exosomal Fn binding, which paralleled the reduction in both exosomal annexins A2 and A6. This reduction in TM exosomal annexins is likely due to an accumulation of annexins A2 and A6 in TM cell membrane following Dex treatment[44]. Second, we show that addition of purified heparan sulfate increased the affinity of TM exosomes for Fn. Third, we removed exosome bound Fn by enzymatically cleaving heparan sulfate. Together this demonstrates TM-derived exosomes bind fibronectin through a mechanism similar to but distinctly different than the syndecan-1-mediated mechanism used by myeloma cell-derived exosomes[17].

The exact role of exosomes in cell-matrix interactions and degradation is unclear; however exosomes from a number of sources have been shown to possess proteolytic activity crucial in degrading the ECM. The membrane bound MT1-MMP has been detected on exosomes [45] and was able to cleave collagen 1, gelatin and pro-MMP2 into its active form [46]. MMP activity is critically important for maintaining outflow facility and therefore TM function [11]. Unexpectedly, we were unable to detect MMP activity attributable to MMP2 or 9. Instead TM exosomes possessed DPP4 activity and protein [16]. DPP4 is a serine protease that cleaves the two N-terminal amino acids from proteins when the second amino acid is proline or alanine (or possibly serine) [47]. It has a transmembrane region but can be cleaved to a soluble form [48]. The total number of physiological substrates for DPP4 is unknown but it has been proposed that they may be extensive [49], and include signaling molecules known to affect TM function/outflow facility such as BMP4 [50], IL-1 [11, 36], bFGF [51], CCL2/MCP-1 [52] and bradykinin [53]. DPP4-truncated signaling molecules were shown to have reduced receptor affinity and efficacy compared to their full-length counterparts [54]. DPP4 has also been shown to possess a number of extra-enzymatic properties, such as binding Fn [55] and mediating virus uptake [56]. Although the significance of exosomal DPP4 is unknown, DPP4 has been localized to invadopodia, affects cell migration/invasiveness [37, 38, 57] and is involved in cell adhesion to Fn [55]. In this context, the reduction in exosomal DPP4 activity from Dex treated TM explants may further reflect the general dysfunction in TM ECM homeostasis that results from Dex treatment. The data presented here is the first description of DPP4 activity in the TM. Future studies will have to determine what role, if any, DPP4 plays in TM cell function and outflow facility/IOP via its enzymatic or extra-enzymatic properties.

Corticosteroids have a number of pathological effects on the TM. For example, Dex decreases phagocytic activity of TM cells [58, 59], increases the secretion of a number of ECM components [21, 60, 61], inhibits TM cell migration and alters the actin cytoskeleton [62]. Interestingly, exosomes may be linked to these processes, such as cell migration [13, 18, 20]. Further, since the formation of invadosome structures is dependent on coordinated actin rearrangement [10], the Dex-induced cross-linked actin networks (CLANs) may inhibit the formation of invadosomes and thus alter exosome release. While we chose to use Dex to model the TM dysfunctions that lead to decreased aqueous humor drainage, increased IOP and glaucoma, elevated levels of the cytokine TGFβ2 in aqueous humor have also been linked to pathological changes in the TM and glaucoma [50, 63]. Not surprisingly, the global effects of TGFβ2 on the TM are similar to those of Dex. They include formation of CLANs, increased secretion of ECM components and accumulation of ECM material in the TM [64–67]. Since exosomes are localized to invadopodia and promote their formation [12, 13], the observed TGFβ2-induced increase in matrix degradation at TM invadosomes [9] suggests the relationship between TGFβ2 and exosomes warrants further investigation.

Our results focused on the dex-induced reduction of exosomal fibronectin affinity by identifying a binding mechanism, a heparin/heparan sulfate bridge, and a candidate heparin/heparan sulfate binding exosomal protein, annexin A2. This protein had previously been identified as highly enriched in TM exosomes along with two other exosomal proteins we examined here, annexin A6 and DPP4[16]. While our data shows that Dex treatment results in decreases in both annexins and DPP4 activity on TM explant exosomes, it is likely the effects of Dex on exosomal protein composition is not limited to these proteins. Since Dex works primarily by activation of glucocorticoid receptors, its effects on cell physiology are broad[68]. Future studies will have to determine what other changes in exosomal protein composition, and the resulting exosomal properties, stem from Dex treatment.

In summary, our data shows for the first time that the human TM explants release exosomes. Moreover, we add to the list of effects corticosteroids have on the TM, showing that Dex treatment causes changes in exosomal cargo and properties. Our results connect the hallmark change in glaucomatous TM tissue, ECM accumulation, with the role of exosomes in cell-matrix interactions. We suspect this connection is not limited to Dex-induced changes in TM. Therefore, we hypothesize that changes in exosome cargo and properties underlie many forms of POAG. Our future studies will test this hypothesis by examining whether other models of glaucomatous TM dysfunction, such as TGFβ2 treatment, also produce pathological changes in exosomal cargo and function.
